# Higher evolutionary rates in life-history traits in insular than in mainland palms

**DOI:** 10.1038/s41598-020-78267-5

**Published:** 2020-12-03

**Authors:** Cibele Cássia-Silva, Cíntia G. Freitas, Larissa Pereira Lemes, Gustavo Brant Paterno, Priscila A. Dias, Christine D. Bacon, Rosane G. Collevatti

**Affiliations:** 1 Laboratório de Genética & Biodiversidade, Instituto de Ciências Biológicas, Universidade Federal de Goiás, Goiânia, GO 74001-970 Brazil; 2 Pós-Graduação em Ecologia e Conservação, Setor de Ciências Biológicas, Universidade Federal do Paraná, Curitiba, PR 81531-990 Brazil; 3 Laboratório de Ecologia Teórica e Síntese, Departamento de Ecologia, Instituto de Ciências Biológicas, Universidade Federal de Goiás, Goiânia, GO 74001-970 Brazil; 4 Departamento de Ecologia, Universidade Federal do Rio Grande do Norte, Natal, RN 59072-970 Brazil; 5 Chair of Restoration Ecology, School of Life Sciences, Technical University of Munich, Emil-Ramann-Str. 6, 85354 Freising, Germany; 6 Department of Biological and Environmental Sciences, University of Gothenburg, Box 461, 405 30 Gothenburg, Sweden; 7 Gothenburg Global Biodiversity Centre, Box 461, 405 30 Gothenburg, Sweden

**Keywords:** Evolutionary ecology, Biogeography, Ecology, Evolution, Plant sciences, Ecology

## Abstract

Isolated islands, due to the reduced interspecific competition compared to mainland habitats, present ecological opportunities for colonizing lineages. As a consequence, island lineages may be expected to experience higher rates of trait evolution than mainland lineages. However, island effects on key life-history traits of vascular plants remain underexplored at broad spatiotemporal scales, even for emblematic island clades such as palms. Here, we used phylogenetic comparative methods to evaluate potential differences in size and macroevolutionary patterns of height and fruit diameter among mainland, continental, and volcanic island palms. Further, phylogenetic beta-diversity was used to determine if lineage turnover supported an adaptive radiation scenario on volcanic islands. Volcanic island palms were taller than their continental island and mainland counterparts, whereas continental island palms exhibited smaller fruit size. Height and fruit size of palms evolved under evolutionary constraints towards an optimal value. However, scenarios of adaptive radiation and niche conservatism were not supported for the height and fruit size of volcanic and mainland palm clades, respectively, as expected. Instead, continental island palms exhibited higher evolutionary rates for height and fruit size. Insular palm assemblages (continental and volcanic) are composed of unique lineages. Beyond representing evolutionary sources of new palm lineages, our results demonstrate that insular habitats are important in shaping palm trait diversity. Also, the higher phenotypic evolutionary rates of continental island palms suggest disparate selection pressures on this habitat type, which can be an important driver of trait diversification over time. Taken together, these results stress the importance of insular habitats for conservation of functional, phylogenetic, and taxonomic diversity of palms.

## Introduction

Oceanic islands are important contributors to generating Earth’s biodiversity. Much of the present-day biodiversity has been generated through pulses of adaptive radiations^1^, as a result of key innovations and/or ecological opportunities, and islands are an important evolutionary arena^1,2^. Adaptive radiation on oceanic islands is often attributed to ecological opportunity, i.e. the exploitation of resources underused by competing taxa^2,3^. Ecological opportunity is mainly a consequence of dispersal and habitat filters, because only a subset of species is able to colonize and survive^4,5^. Those species that overcome initial filters and establish in depauperate island communities can diversify widely, filling vacant niche spaces^4,6^, and frequently have distinctive trait diversity compared with the mainland source^1^. However, the role of oceanic island colonization on species trait evolution and divergence patterns remains largely underexplored, especially for plants^7,8^.

Plants in insular ecosystems experience unique eco-evolutionary trajectories that are related to their geographic isolation, which directly impacts trait evolution^7,9^. Under the commonly expected scenario of adaptive radiation on isolated islands^10^, trait evolution is predicted to follow an ‘early-burst’ then a ‘slow-down’, i.e. trait evolution is initially fast and then slows down^11^ as vacant ecological niches are filled and competition increases^4,12^. In contrast, trait evolution of mainland assemblages may be constrained due to the higher diversity of co-distributed species^1,13^. The traits of mainland species that originally facilitated lineage diversification by allowing for coexistence may be maintained through time, i.e. through phylogenetic niche conservatism^14–17^.

For plants, a wide range of ecological strategies and responses to competition, stress, and disturbance are mediated by life-history traits such as height and seed size^18^. Plant height is related to competitive ability because it determines plant survival in varying environmental conditions and biotic interactions^19,20^; it is also a proxy for generation time^19^. Variation in seed size is correlated to dispersal ability and establishment opportunity, being a good measure of seedling capacity to survive to adverse conditions^18^. Therefore, taken together, plant height and seed size can identify the role of ecological opportunity and the distinct selective forces that shape the survival strategies of plants in contrasting habitats, such as on the mainland and on islands^8,18,21^, even in phenotypically diverse plant families like the palms (Arecaceae).

The species-rich palm family, with ca. 2600 species^22^, has a wide diversity of growth forms ranging from small acaulescent shrubs to lianas and canopy-emergent species^23,24^. They also present a great diversity of fruit form, color, and size^23,25^. As most palms are single-seeded, fruit size is correlated with seed size^26,27^. Fruit size is thus an important trait to understand the role of evolutionary and ecological processes shaping palm diversification and distribution patterns, including on isolated islands^26,28,29^.

Palms are an emblematic group of tropical islands^30,31^, and fruit size is suggested to be an important trait for palm colonization and the diversification process on isolated islands^28,29^. Recent findings show that shifts towards smaller fruits favored long-distance seed dispersal by small frugivores, allowing the colonization of isolated islands by palms^29^. Additionally, speciation rates of small fruited palms were shown to be higher on remote islands^28^. These findings raise the question of whether these isolation-driven processes also lead to distinct evolutionary patterns in height and fruit size on volcanic islands (i.e. islands that never been connected to continental landmasses^32^), when compared to continental islands (i.e. islands that once were connected to mainland shelf^32^), and especially to their mainland counterparts.

Continental and volcanic islands exhibit markedly environmental differences in precipitation and elevation^32^, which are important environmental determinants of trait distribution patterns in palms^33^. The present-day patterns in palm species diversity also differ between continental and volcanic islands, since spectacular insular radiations of palms occurred mainly on continental islands (e.g. Cuba, Madagascar and New Caledonia)^30^. Here, we test for potential differences in plant size and macroevolutionary patterns of height and fruit diameter among mainland, continental island, and volcanic island palms, using volcanic islands as a proxy for high geographic isolation^32^.

Decreases in fruit size are linked to dispersal and diversification of palms on volcanic islands^28^. Due to the allometric relationship between plant height and fruit size^34^, we hypothesize that (1) palm assemblages from volcanic islands have smaller height and smaller fruit size than mainland and continental island assemblages. Further, we hypothesize that (2) palm life-history traits on mainland and continental islands evolve under evolutionary constraints, such as niche conservatism. Since adaptive radiation refers to an increase in diversification and morphological rates, as well as morphological disparity^1,10^, we hypothesize that on volcanic islands (3) palm life-history traits evolve under an early-burst pattern and exhibit higher evolutionary rates consistent with adaptive radiation scenario, as driven by ecological opportunities and the filling of vacant niche space on isolated islands^1,11^.

Owing to the in situ adaptative radiation expected for palm trait evolution in volcanic islands and the suggestion that volcanic islands trigger palm speciation^28^, we expect phylogenetic clustering in this habitat type. We also predict that (4) volcanic island assemblages are composed of unique lineages rather than being a subset of continental and mainland assemblages. We test all hypotheses globally as well as separately for the Afrotropics, Australasia/IndoMalaya, and Neotropics, owing to the differences in the historical contingency in these distinct biogeographical realms and their potential imprints in palm trait diversity and lineage composition^30,35,36^. Here, we investigate the role of the mainland and insular (continental and volcanic) habitats on functional and phylogenetic palm diversity, which can identify the importance of these habitats in the conservation of different facets of palm diversity, such as functional, phylogenetic, and taxonomic diversity.

## Results

### Quantitative patterns in palm life-history traits

**Figure 1:**
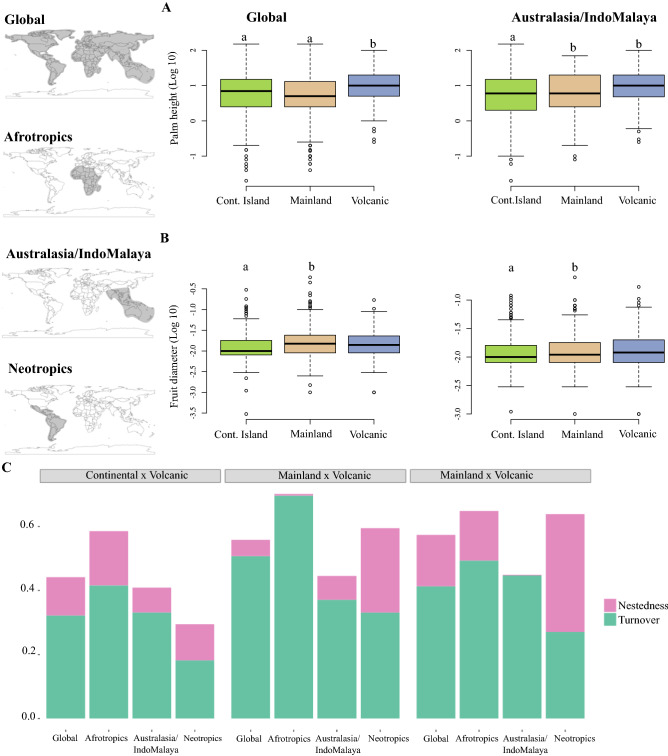
The multiple comparisons of palm height (**A**) and fruit diameter (**B**) among habitat types at the global scale and on Australasia/IndoMalaya, since the differences were not significant on Afrotropics (Height: F_2,246_:0.421, p = 0.656; Fruit size: F_2,190_:0.106, p = 0.899) and Neotropics (Height: F_2,643_:1.833, p = 0.161; Fruit size: F_2,628_:0.132, p = 0.876). The mainland is shown in beige, continental islands in light green, and volcanic islands in blue. Groups followed by different letters are significantly different according to the Tukeys’ post hoc honest significant difference (p < 0.001). In (**C**) the phylogenetic beta-diversity (PBD) measured with the Phylosor dissimilarity index, and their resultant components, where nestedness is shown in pink and turnover in green. Continental and volcanic refers to island categories. PBD_Continental × Volcanic_ = 0.441_Global_; 0.585_Afrotropics_; 0.408_Australasia/IndoMalaya_; 0.295_Neotropics_. PBD_Mainland × Continental_ = 0.558_Global_; 0.701_Afrotropics_; 0.445_Australasia/IndoMalaya_; 0.594_Neotropics_. PBD_Mainland × Volcanic_ = 0.573_Global_; 0.648_Afrotropics_; 0.448_Australasia/IndoMalaya_; 0.638_Neotropics_. The different biogeographical realms (Afrotropics, Australasia/IndoMalaya, and Neotropics) are shown on the left.

Out of 2,539 species in the palm phylogeny^37^, we obtained height data for 1982 (78%) and fruit diameter data for 1687 (66%) species (Supplementary Appendix S1). Using the maximum clade credibility (MCC) palm tree together with trait data in Phylogenetic Generalized Least Squares (pGLS) models, we found that palms from volcanic islands were on average taller than their continental island and mainland counterparts at global scale (F_2;1734_: 39.06, p < 0.001, n = 1737), and in the Australasia/IndoMalaya biogeographical realm (F_2;834_: 50.64, p < 0.001, n = 837; Fig. 1A, Supplementary Table S1). Palms from continental islands had on average smaller fruit size than their mainland counterparts at global scale (F_2,1472_: 4.711, p < 0.001, n = 1475) and in the Australasia/IndoMalaya biogeographical realm (F_2,644_: 7.06, p < 0.001, n = 647; Fig. 1B, Supplementary Table S1).

The differences in palm height and fruit size among different habitats were not significant in the Afrotropics (Height: F_2,246_: 0.421, p = 0.656, n = 249; Fruit size: F_2,190_: 0.106, p = 0.899, n = 193) or in the Neotropics (Height: F_2,643_: 1.833, p = 0.161, n = 646; Fruit size: F_2,628_: 0.132, p = 0.876, n = 631). The interaction between height and habitat type to explain the differences in palm fruit size was also not significant (F_2,1309_: 0.531, p = 0.531, n = 1315). However, these pGLS models for palm height and fruit size as a function of habitat type were sensitive to phylogenetic uncertainty (Figs. S1, S2), where model estimates varied across 100 trees randomly sampled from the posterior distribution^37^.

### Evolutionary patterns in palm life-history traits

**Figure 2:**
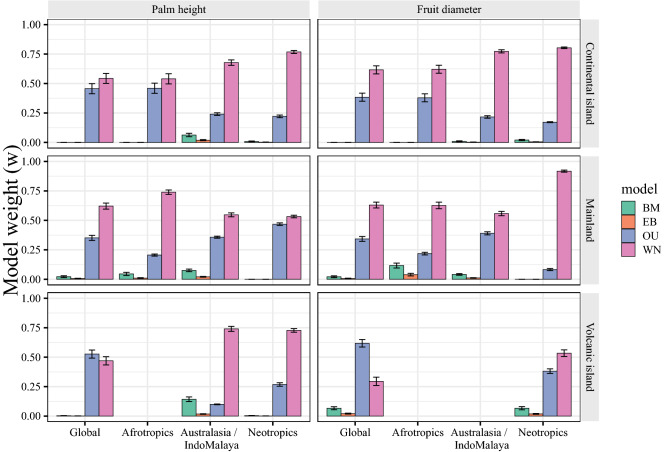
Comparative evolutionary model fit of palm height and fruit size across 100 random trees for tribes and subtribes representing habitat types worldwide and within biogeographical realms (Afrotropics, Australasia/IndoMalaya, and Neotropics). *BM* brownian-motion, *EB* early-burst, *WN* white-noise, *OU* Ornstein–Uhlenbeck model. Model weight was calculated from sample-size corrected AIC (AICc) for each evolutionary model across the 100 trees. Error bars represent standard error of the mean.

Palm height was most consistent with an Ornstein–Uhlenbeck (OU) model, which describes evolution under stabilizing selection (Table 1). Using representative clades of each habitat type at the global scale (tribe and subtribe level) and within each biogeographical realm (genus level; see Fig. S3), we found that palm height fit an OU model only for Ptychospermatinae, representing the volcanic islands at the global scale (Fig. 2). While for Dypsidinae and Iriarteeae, representing the continental islands and mainland respectively (Fig. S3), the White-Noise (WN) model that describes variation as independent of phylogeny, was the best-fit model (Fig. 2). For representative genera within biogeographical realms, the WN was the best-supported model for palm height, with the exception of *Dypsis*, representing the continental island of Afrotropics, in which the OU model was supported (Fig. 2). We found an increase in the evolutionary rate for palm height on continental islands compared to patterns across the entire phylogeny (Table 2). Similarly, in pairwise comparisons, mainland and volcanic island palms exhibited a decrease in their evolutionary rates for palm height compared to continental island assemblages (Table 2).

**Table 1:** Comparative evolutionary model fit of palm height (n = 1,982) and fruit size (n = 1,687) using the MCC palm tree.

Trait	Model	∆AICc	log-likelihood	n	AICc	AIC_w_
Height	**OU**	**0**	** − 1468.971**	**3**	**2943.955**	**1**
	WN	165.238	** − **1552.594	2	3109.194	0
	BM	1161.223	** − **2050.586	2	4105.179	0
	EB	1163.237	** − **2050.59	3	4107.193	0
Fruit size	**OU**	**0**	** − 490.578**	**3**	**987.172**	**1**
	WN	89.750	** − **536.457	2	1076.923	0
	BM	854.343	** − **918.754	2	1841.516	0
	EB	856.358	** − **918.757	3	1843.530	0

*BM* brownian-motion, *EB* early-burst, *WN* White Noise, *OU* Ornstein–Uhlenbeck model, *n* represents the number of parameters of each model.

The weighted Akaike information criterion (AIC_w_) was used to evaluate model fit.

In bold, the best-fit model (AIC_w_ = 1 and ΔAIC_c_ ≤ 2).

**Table 2:** Evolutionary rate differences in palm height (n = 1737) and fruit size (n = 1475) for each habitat type (mainland, continental and volcanic islands) and for both entire phylogeny and pairs of habitats.

Trait	Habitat type			Pairwise comparisons		
	Mainland	Continental Island	Volcanic Island	Mainland–Cont. Island	Mainland–Volcanic Island	Volcanic–Cont. Island
**Height**						
Rate difference (p)	− 0.036 (0.039)	0.064 (0.999)^+^	− 0.044 (0.050)	− 0.059 (0.005)^−^	− 0.020 (0.259)	− 0.079 (0.003)^−^
**Fruit size**						
Rate difference (p)	− 0.025 (0.036)	0.046 (0.999)^+^	− 0.036 (0.032)	− 0.042 (0.002)^−^	− 0.021 (0.163)	− 0.063 (0.002) ^-^

p > 0.975^**+**^ implies on increased rates associated with the habitat type and p < 0.025^−^ implies on decreased rates. p > 0.025 and < 0.975 mean no trait evolution rate difference between the habitat type and the entire phylogeny or in pairwise comparisons.

^**+**^Increase on trait evolutionary rates.

^−^Decrease on trait evolutionary rates.

Palm fruit size fit an OU model of evolution (Table 1), as well as for the Ptychospermatinae representing volcanic islands at the global scale (Fig. 2). For clades representing continental islands (Dypsidinae) and the mainland (Iriarteeae) at the global scale (see Fig. S4), as well as for all genera representing distinct habitats types within biogeographical realms, the WN was the best-supported model for palm fruit size (Fig. 2). We found an increase in the evolutionary rate for palm fruit size on continental islands compared to patterns across the entire phylogeny (Table 2). Also, we found a decrease in evolutionary rates for palm from volcanic islands compared to continental islands (Table 2).

### Lineage composition patterns

The high phylogenetic dissimilarity among ecosystems was driven by the turnover component of beta-diversity at both global and biogeographical scales (Fig. 1C). The exception was for the Neotropical mainland and volcanic islands, where nestedness explained the phylogenetic dissimilarity. Overall, higher values of lineage turnover were found between mainland and continental islands, whereas lower turnover values were found between island categories (Fig. 1C).

## Discussion

Life-history traits play a key role in the colonization and diversification on islands, especially those that are related to resource acquisition, space occupancy and dispersal ability, such as plant height and fruit size^7,28^. We found quantitative differences in palm height and fruit size among mainland, continental and volcanic islands habitat types (hypothesis 1). Volcanic island palms are on average taller than their continental islands and mainland counterparts globally and in Australasia/IndoMalaya (Fig. 1A). Also, continental island palms exhibit smaller fruit sizes than mainland ones (Fig. 1B). Macroevolutionary models revealed no difference in trait evolution between mainland and insular palm clades (hypotheses 2 and 3). Palm height and fruit size evolve under evolutionary constraint suggesting niche conservatism, but a constrained evolutionary pattern was not identified for mainland and continental island palm clades. Although insular palm assemblages are the result of diversification of unique lineages (hypothesis 4; Fig. 1C), a scenario of adaptive radiation of trait evolution and higher phenotypic evolutionary rates was not found for volcanic islands palms. In contrast, palms from continental islands exhibited higher phenotypic evolutionary rates.

We found that palms from volcanic islands were significantly taller than palms from other habitat types (continental islands and mainland). This finding is in line with previous studies that ‘gigantism’ is a more common evolutionary pathway on isolated island plants than ‘dwarfism’^6,8^, i.e. smaller height as we expected following our hypothesis 1. Size shifts towards taller plants is suggested to be the result of selection for increased longevity on isolated islands^6,8^, such as the volcanic ones, because plant height is a proxy of longevity^19^. Further, the release from interspecific competition might also be responsible for the increase in plant height and longevity on isolated islands, because in general isolated islands exhibit lower species diversity, but higher species abundances (higher intraspecific competition) compared with plant assemblages in mainland^8^.

The intense intraspecific competition on isolated islands due to higher population densities could also favor the production of larger seeds^8,21^. Large seeds and consequently large fruits in palms tend to be more competitive since their seedlings have a higher likelihood of survival compared to smaller seeded species^8,21,33^. Strong selection towards increasing survival may overcome the selective pressure to increase dispersal capacity, leading to an increase in fruit and seed sizes. This selection contradicts our prediction of finding smaller fruits on isolated volcanic islands (hypothesis 1), which was based on previous results linking smaller fruits to a higher probability of colonization of volcanic islands in the Afrotropics and Australasia/IndoMalaya^28^. However, large seed (and therefore fruit) size is not a hinderance to recurrent oceanic dispersal and island colonization events for palms (Borasseae)^34^.

Palms on volcanic islands in the Afrotropics and Australasian/IndoMalayan biogeographical realms exhibit higher speciation rates^28^, suggesting high diversification in these habitats^10^. Island colonization is expected to follow a pattern of adaptive radiation with explosive lineage diversification and trait disparity, followed by a slow-down^3,11,38^. However, this prediction was not supported for the traits analyzed here in palm clades from volcanic islands. The early-burst model was not supported for either height or fruit size for the volcanic island palm clades (hypothesis 3). An early-burst pattern in species traits evolution actually appears to be empirically rare across the Tree of Life^11,39,40^. Extinction tends to erase signals from the molecular phylogenies, which can obscure the signal of early, rapid diversification followed by slows down in phylogenies^41,42^. Also, here, some clades representing ‘volcanic islands’ are not endemic to volcanic islands, such as the Ptychospermatinae clade (Figs. S3, S4), which can make it even more difficult to identify early-burst patterns. Palms from volcanic islands also did not have higher evolutionary rates for height and fruit size, but instead had similar and lower rates compared with the mainland and continental islands, respectively.

Overall, our findings show that the high diversification of isolated island palms^28,30^ is decoupled from high phenotypic evolutionary rates. In radiations driven by ecological opportunity, increased diversification rates can indeed precede an increase in trait evolution^43^. However, volcanic island palms had significant trait divergence (plant height), indicating adaptive radiation in those habitats through morphological and/or functional disparity^10^. Also, we found increases in trait evolutionary rates for continental islands. Thus, contrary to evidence for mammals, in which evolutionary rates of body size did not differ between islands and mainland species^44^, here we cannot fully exclude the effects of island colonization on palm trait evolution.

Outstanding palm radiations also occurred on large continental islands^30,31^, such as *Coccothrinax* on Cuba and *Dypsis* on Madagascar. Indeed, we found that insular (continental and volcanic) palm flora assembly was driven by lineage turnover, suggesting in situ speciation. Throughout palm evolutionary history, continental islands most likely acted as important sources for new lineages by feeding colonization from islands back to the adjacent mainland and/or to volcanic islands, as evidenced in other ancient plant clades^45^. Also, here we demonstrate the importance of continental islands as arenas for trait evolution. Together, the quantitative differentiation in fruit size and higher phenotypic evolutionary rates on continental islands indicate disparate selective pressures when compared to mainland and volcanic islands. This differential selective pressure is striking because there is evidence for gene flow between continental islands and the mainland through geological time^28^, largely due to sea-level changes across time. Major sea level changes occurred during the time the palm family originated and diversified^35,36^ (ca. 100 Ma and ca. 70–7 Ma, respectively). Such disparate selection pressure on continental islands can be an important driver of trait diversity^8,21^.

Undoubtedly, insular habitats, especially the continental ones, have an important role in generating palm species and lineage diversity^31,46^. Beyond being an important source of new palm lineages and species, our results demonstrate that insular (continental and volcanic) habitats are important in shaping palm trait diversity, as evidenced by the trait divergence on continental (fruit size) and volcanic (height size) palms traits. The evolutionary importance of these ecosystems stresses their role in the conservation of different facets of palm diversity (i.e. functional, phylogenetic, and taxonomic).

## Methods

### Palm ecosystem type and phylogenetic data

We obtained a list of accepted palm species from the World Checklist of Selected Plant Families (http://apps.kew.org/wcsp accessed on July 15, 2018). In this dataset, palm species records are described across the world within geographic units categorized as ‘botanical countries’ as defined by the International Working Group on Taxonomic Databases (TDWG), which split large countries (e.g. Australia, Brazil, China, and the USA) into states or provinces. Then, to all TDWG level 3 units records for palms, we categorized them by island or mainland habitat types^46^. We defined islands as landmasses smaller than Australia surrounded by an ocean and included both groups of islands or single islands^32,46^.

Based on the TDWG level 3 units, we further assigned palm species into three habitat type categories: mainland, continental island, and volcanic island, based on their geographic ranges, where species occupied more than one category (i.e. widespread species) were omitted from the main analyses (except for model selection analyses, where all species of a given clade were included, see below). This classification of species according to their habitat type was made at a global scale and within biogeographical realms separately. We assigned each species to one of three biogeographical realms (i.e. Afrotropics, Australasia/IndoMalaya, and Neotropics) defined by Olson et al.^47^, but here we combined the Australasia/Oceania with IndoMalaya (hereafter, Australasia/IndoMalaya) because of their shared island palm flora^48^. We included the Hawaiian palm flora in the Neotropics biogeographical realm due to the Neotropical ancestry of several Hawaiian angiosperms^49^, including the only lineage of native palms^50^. Lastly, we obtained information on island geology (continental and volcanic) from the United Nations Environment Programme Island Directory (http://islands.unep.ch/isldir.htm/ accessed in August 2018).

We used a species-level palm phylogeny^37^ based on the taxonomy from the Govaerts et al.^51^ that includes 2539 species. Analyses were performed either on maximum clade credibility tree (MCC)^52^ or on a set of 100 trees randomly sampled from the posterior distribution provided in^37^.

### Palm height and fruit size data

We obtained maximum values of palm stem height (height, hereafter) from the PalmTraits 1.0 dataset^53^. Here, we followed Henderson^25^ and used fruit diameter to represent palm fruit size. We compiled fruit diameter data from the primary literature (data sources are provided in Supplementary Table S2), the global database of plant traits^54^, and palm websites (http://palmweb.org and http://www.palmpedia.net). Height and fruit diameter were log_10_-transformed prior to analysis to improve data normality following Rueda et al.^17^_._ All analyses were carried out in R v. 3.4.3^55^.

### Quantitative patterns in palm life-history traits

To test hypothesis 1 that palm assemblages from volcanic islands have smaller height and fruit size than mainland and continental islands assemblages, we built phylogenetic Generalized Least Squares (pGLS) models for each trait using the *corBrownian* covariance matrix in “caper”^56^. We built pGLS models at global scale and for each biogeographical realm separately using the MCC tree. Tukey’s post hoc honest significant difference (HSD) tests were performed on the significant results of the pGLS models using the *glht* function implemented in “multcomp”^57^. To evaluate the role of phylogenetic uncertainty in our pGLS results, we performed sensitivity analyses following the approach implemented in “sensiPhy”^58^, by fitting each pGLS model across 100 random phylogenies sampled from the posterior distribution^37^.

As we found a significant correlation between palm fruit diameter and palm height for species where information on both traits was available (r =  − 0.844, df = 1514, SE = 0.315, p < 0.001, n = 1516; Supplementary Fig. S5) using the best-fitted covariance matrix for data (Supplementary Table S3), we also performed a pGLS model to access the differences in fruit size as a function of habitat type taking in account the height as interaction factor.

### Macroevolutionary model selection

We evaluated the evolutionary mode of palm life-history traits through model selection using the MCC palm tree. Further, to test the hypothesis about macroevolutionary trait patterns on continental islands and the mainland (i.e. under phylogenetic niche conservatism, hypothesis 2), as well on volcanic islands (i.e. under an adaptive radiation scenario, hypothesis 3), we selected representative clades of each habitat type (tribe and subtribe level) and within each biogeographical realm (genus level), see Table 3. Here, all species of clades (i.e. including widespread ones) were included in analyses (see Fig. S3). To avoid potential bias due to differences in clade ages, all clades were selected from the Arecoideae subfamily and each clade had at least 10 species with height and/or fruit size data for model selection^11^.

**Table 3:** Tribes (subtribes) and genera (clades) that were chosen to represent the study habitat type at global scale and within biogeographical realms (Afrotropics, Australasia/IndoMalaya, and Neotropics), respectively.

	Habitat type	Clade Height (n) − Fruit size (n)	Description*
Global	Continental Islands	Dypsidinae (n = 167) (158) − (110)	The subtribe is confined primarily to Madagascar (a continental island), but with outliers in eastern Africa and islands of the Indian ocean
	Mainland	Iriarteeae (n = 32) (31) − (31)	The tribe is confined to the Neotropical mainland, especially in South America
	Volcanic Islands	Ptychospermatinae (n = 67) (53) − (44)	This subtribe is widespread in volcanic islands and atolls (e.g. Caroline, Fiji, Mollucas, Samoa, Solomon, and some Philippines islands)**. However, some genera such as the *Ptychosperma* occurs primarily in the New Guinea (a continental island)**, as well as in East Malesia, Australia
Afrotropics	Continental Islands	*Dypsis* (n = 162) (155) − (105)	The genus is confined primarily to Madagascar
	Mainland	*Raphia* (n = 20) (17) − (17)	The genus is confined primarily to the African mainland
	Volcanic Islands	na	
Australasia/IndoMalaya	Continental Islands	*Calyptrocalyx* (n = 26) (26) − (25)	The genus is confined primarily to New Guinea
	Mainland	*Livistona* (n = 28) (26) − (26)	The great diversity of species occurs in Australia, but the genus is widespread in the Australasia realm including some islands, such as New Guinea and Solomon, as well as an outlier on Africa (*Livistonia carinensis*)
	Volcanic Islands	*Clinostigma* (n = 10) (10) − na	The genus is widespread throughout the volcanic islands and atolls** from the Pacific (e.g. Caroline, Fiji, New Ireland, Samoa, Solomon, Vanuatu)
Neotropics	Continental Islands	*Coccothrinax* (n = 51) (27) − (17)	Species ranging from south Florida to Colombia, but the majority of species are confined to Cuba (a continental island)**
	Mainland	*Chamaedorea* (n = 106) (101) − (84)	Species are confined to the Neotropics mainland ranging from central Mexico to Brazil and Bolivia
	Volcanic Islands	*Pritchardia* (n = 28) (26) − (26)	Species are confined primarily to Hawaii

Height: Maximum stem height (m); Fruit size: fruit diameter (m), n: the total number of species from clade^51^ and with trait (height and fruit size) data, na: lack of sufficient data {i.e. lack of clades representing a given habitat type (Afrotropics volcanic island) or less than 10 species with available fruit size data (*Clinostigma*).

*Dransfield et al.^23^, Essig^59^.

**UNEP (http://islands.unep.ch/isldir.htm).

We compared the fit of four different models of trait evolution. The Brownian-Motion (BM) model assumes that trait variance accumulates with time and thus species divergence is proportional to time^60^. The Early-Burst (EB) model states that trait evolution is faster early in the history of a clade and decreases gradually over time, consistent with a scenario of adaptive radiation^11^. The Ornstein–Uhlenbeck (OU) model describes constrained trait evolution^61^ and can indicate phylogenetic niche conservatism given that trait evolution is constrained towards an optimal value^16,17,62,63^. Lastly, the White-Noise (WN) model assumes that trait evolution occurs so fast that all traces of shared ancestry are lost, which is equivalent to trait variation being independent of phylogenetic relationships^64^. We built evolutionary models describing height and fruit size using the MCC tree with the *fitContinuous* in “geiger”^65^. Due to the decreased taxonomic resolution of the phylogeny at lower taxonomic levels, such as genus, we built evolutionary models for the clades representing the habitats using 100 random ‘clade trees’ pruned from the posterior distribution of palm phylogeny^37^. To achieve this, we used an extension of *tree_continuous* from “sensiPhy”^58^ to accommodate phylogenetic uncertainty. Model selections were performed using the weighted Akaike information criterion (AIC_w_), which was calculated from sample-size corrected AIC (AICc)^66^.

### Evolutionary rates on islands

To test if volcanic island palms have higher rates of height and fruit size evolution (hypotheses 2), we first estimated the evolutionary rates of both traits on the MCC tree using a phylogenetic ridge regression approach with the *rrphylo* function, considering ancestral trait values, implemented in “RRphylo”^44,67^. Then, we evaluated if evolutionary rates differ depending on where species occur: mainland, continental, and volcanic islands. To achieve this, we used the *search.shift* from “RRphylo”, which allowed us to verify if evolutionary rates associated with a specific habitat type are higher or lower than overall rates across the entire phylogeny. In this approach, a rate shift is identified when the difference between the rates preceding and succeeding a node is significantly different from the difference between the previous and posterior evolutionary rates considering all nodes in the phylogeny and within a 95% confidence interval^44^.

### Biogeographic and lineage composition patterns

To test if volcanic island assemblages are composed of unique lineages (hypothesis 4), we calculated phylogenetic beta-diversity among mainland, continental and volcanic islands for global and within biogeographical realms. To estimate phylogenetic-beta, we used a presence–absence matrix of palms in an additive partitioning framework, portioning phylogenetic beta diversity into turnover and nestedness components^68,69^. We calculated the phylogenetic beta diversity and its components using the Phylosor dissimilarity index^70^ using “betapart”^71^. The index ranges from 0 to 1, where 0 indicates that two habitats have similar lineage composition and 1 indicates that two habitats have different lineage composition^69^.

## Supplementary information

Supplementary Information 1.

Supplementary Information 2.

Supplementary Information 3.

## Data Availability

The palm tree phylogeny used in this paper is publicly available^37^. The palm maximum stem height and fruit size used in all analyses are publicly available in a palm trait database^53^ and provide as Supplementary (Appendix S1), respectively.
