# Supplementary material for: Higher evolutionary rates in life-history traits in insular than in mainland palms
Source: Sci Rep. 2020 Dec 3;10:21125. doi: 10.1038/s41598-020-78267-5 (PMC7713303; doi:10.1038/s41598-020-78267-5)
Supplement: Supplementary file 1 — Supplementary Information 1. [file 41598_2020_78267_MOESM1_ESM.docx]

**Higher evolutionary rates in life-history traits in insular than in mainland palms**

Cibele Cássia-Silva^1*^, Cíntia G. Freitas^2^, Larissa Pereira Lemes^3^, Gustavo Brant Paterno^4,5^, Priscila A. Dias^1^, Christine D. Bacon^6,7^, Rosane G. Collevatti^1^

**Supplementary**

**Appendix S1**. Palm trait (height and fruit diameter), habitat type (mainland, continental and volcanic islands), and biogeographic realm data. Palm species list for height and fruit size data used in the pGLS model and ridge regression approach analyses. Palm species list with paired data (height and fruit size) used in the correlation test. Afrotropics, Australasian/IndoMalayan, and Neotropical palm species list and their respective habitat type data used in the pGLS models [See excel spreadsheet file].

**Appendix S2**. Trait data (height and fruit diameter) of all clades used to represent mainland, continental and volcanic islands globally and within biogeographic realms [See excel spreadsheet file].

**Table S1**. Multiple comparisons of means (Tukey contrasts) for pGLS models for palm height (log _10_) and fruit size (log _10_), considering all possible interactions (linear hypotheses) between habitat types (mainland, continental, and volcanic island) at global scale and in Australasia/IndoMalaya. The table shows the slope (**estimate**) of each regression (**linear hypothesis**) with their standard errors (**Std. Error**) and p-values adjusted for multiple comparisons by the single-step method. In bold, significant comparisons.

| **Height** | **Linear Hypotheses** | **Estimate** | **Std. Error** | **z value** | **Pr(>\|z\|)** |
| --- | --- | --- | --- | --- | --- |
| Global | Mainland - Cont.Island = 0 | 0.028 | 0.032 | 0.887 | 0.644 |
|  | **Volcanic - Cont.Island = 0** | **0.236** | **0.027** | **8.726** | **< 0.001** |
|  | **Volcanic - Mainland = 0** | **0.207** | **0.036** | **5.689** | **< 0.001** |
| Australasia/IndoMalaya | Mainland - Cont.Island = 0 | 0.045 | 0.03 | 1.518 | 0.278 |
|  | **Volcanic - Cont.Island = 0** | **0.250** | **0.024** | **10.011** | **< 0.001** |
|  | **Volcanic - Mainland = 0** | **0.204** | **0.034** | **6.003** | **< 0.001** |
| **Fruit size (diameter)** |  |  |  |  |  |
| Global | **Mainland - Cont.Island = 0** | **0.076** | **0.024** | **3.054** | **< 0.001** |
|  | Volcanic - Cont.Island = 0 | 0.041 | 0.03 | 1.325 | 0.378 |
|  | Volcanic - Mainland = 0 | -0.034 | 0.032 | -1.076 | 0.526 |
| Australasia/IndoMalaya | Mainland - Cont.Island = 0 | **0.082** | **0.022** | **3.735** | **< 0.001** |
|  | Volcanic - Cont.Island = 0 | 0.046 | 0.026 | 1.708 | 0.2 |
|  | Volcanic - Mainland = 0 | -0.036 | 0.028 | -1.29 | 0.398 |

**Figures S1**. Sensitivity analyses of phylogenetic uncertainty on the estimates of phylogenetic generalized linear models (pGLS) of **height** (log_10_) as a function of habitat type. Simulations were performed across 100 alternative phylogenetic trees. Frequency distribution of model estimates and p-values for Continental islands (**AD**), Mainland (**BE**) and Volcanic islands (**CF**). Solid black lines represent the average estimate across all 100 trees.


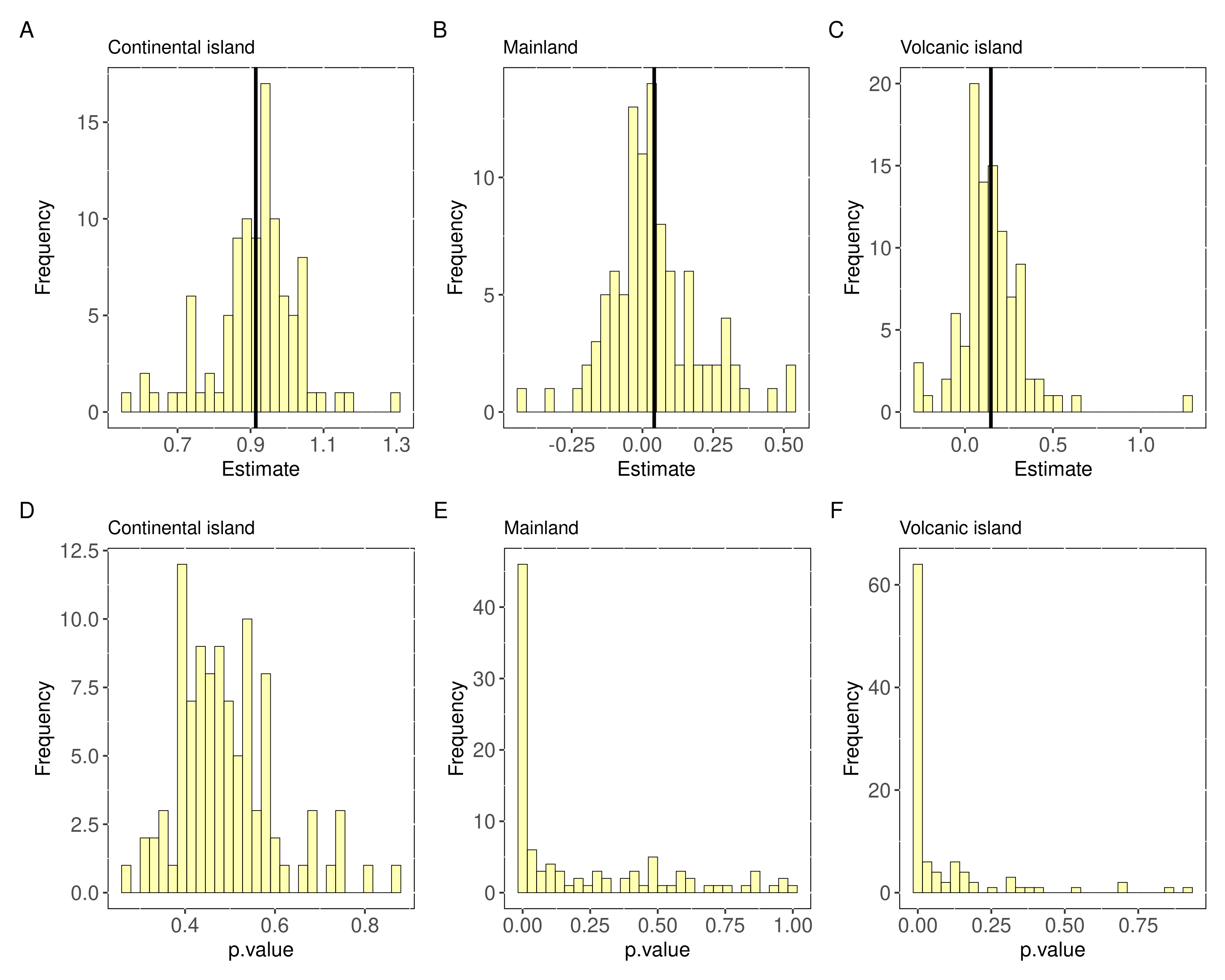


**Figures S2**. Sensitivity analyses of phylogenetic uncertainty on the estimates of phylogenetic generalized linear models (pGLS) of **fruit size** (log_10_) as a function of habitat type. Simulations were performed across 100 alternative phylogenetic trees. Frequency distribution of model estimates and p-values for Continental islands (**AD**), Mainland (**BE**) and Volcanic islands (**CF**). Solid black lines represent the average estimate across all trees.


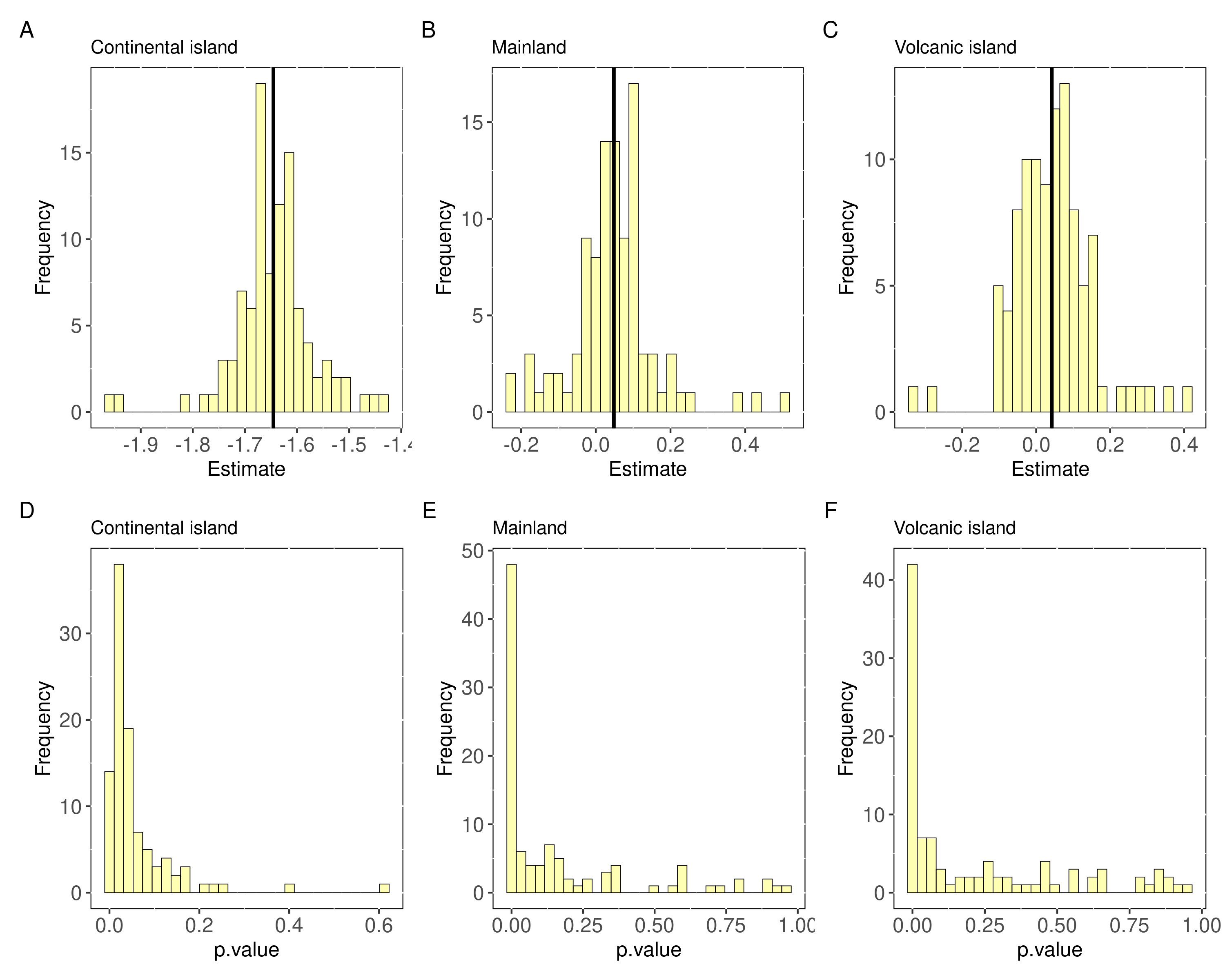


**Figure S3.** Reconstruction of ancestral states for the height of palm clades representing different habitat types, in different biogeographic realms shown in left map. Continental island: green, mainland: beige, and volcanic islands: blue. The ancestral reconstructions were obtained from stochastic character mapping and are shown for one representative maximum clade credibility tree of each clade (out of 100 randomly selected and analyzed phylogenies).


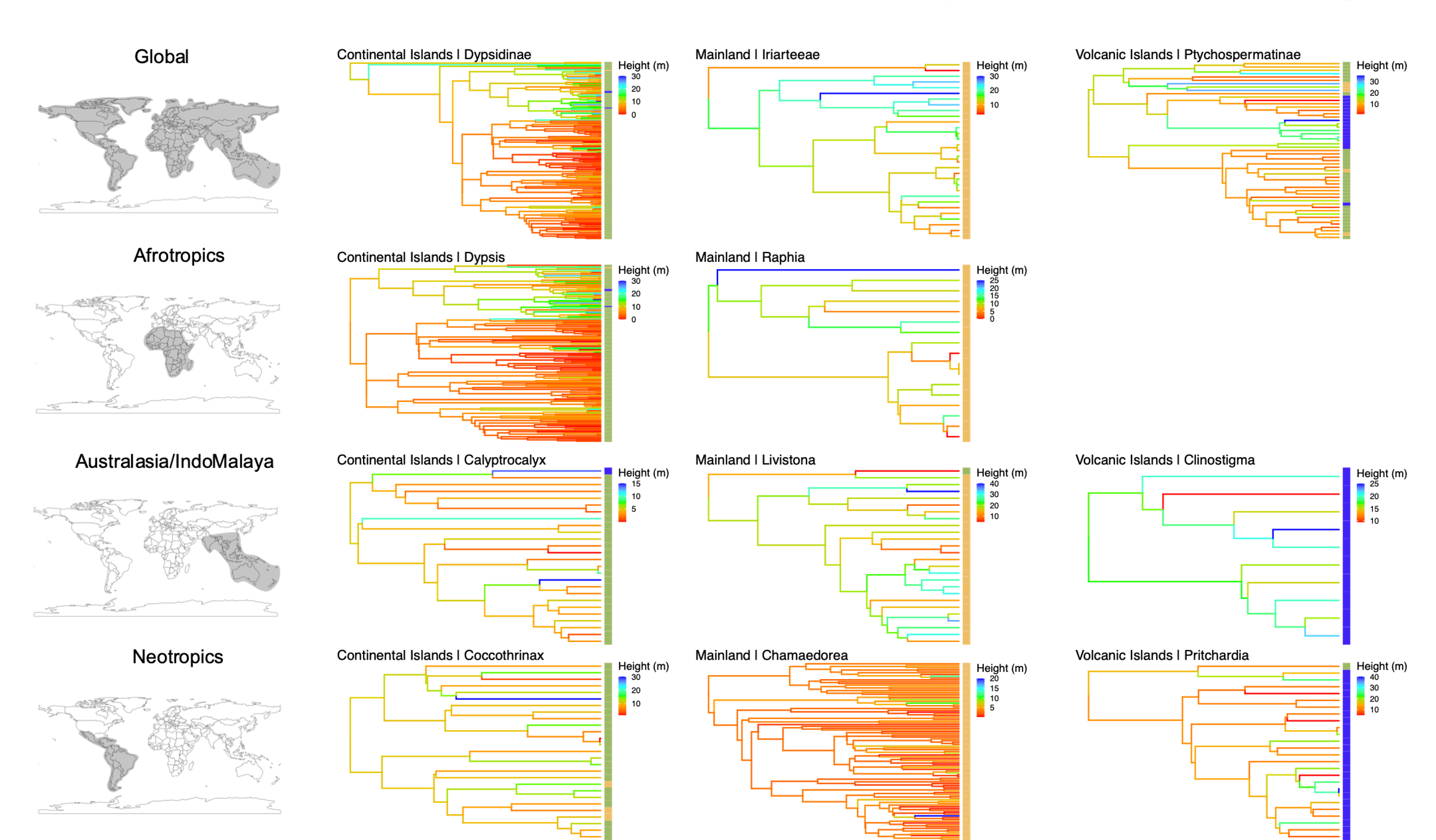


**Figure S4.** Reconstruction of ancestral states for fruit size (diameter - m) of palm clades representing different habitat types, in different biogeographic realms shown in the left map. Continental island: green, mainland: beige, and volcanic islands: blue. The ancestral reconstructions were obtained from stochastic character mapping and are shown for one representative maximum clade credibility tree of each clade (out of 100 randomly selected and analyzed phylogenies).

**
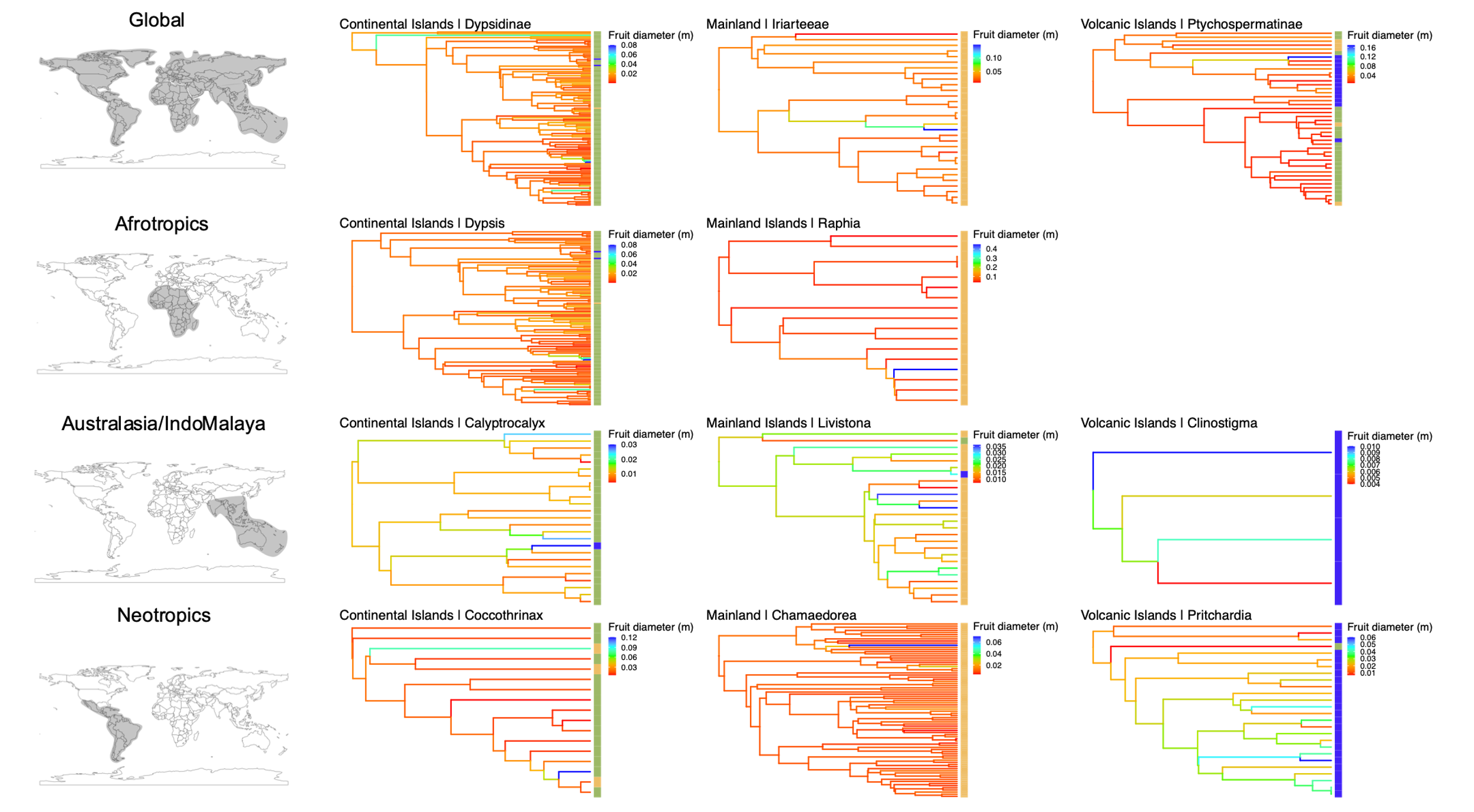
**

**Table S2. Data Sources -** literature sources used to obtain palm fruit size.

Aliaga-Rossel, E. N. Z. O. (2011). Phenology and germination of the Chonta Palm, *Astrocaryum gratum*, in a sub-montane forest. *Palms*, *55*, 84-92.

Bacon, C. D., & Baker, W. J. (2011). *Saribus* resurrected. *Palms*, 55, 109-116.

Banka, R., & Baker, W. J. (2004). A monograph of the genus *Rhopaloblaste* (Arecaceae). *Kew Bulletin*, 47-60. <https://doi.org/10.2307/4111073>

Baker, W. J., & Couvreur, T. L. (2012). Biogeography and distribution patterns of Southeast Asian palms. *Biotic Evolution and Environmental Change in Southeast Asia*, 82, 164.

Baker, W. J., Zona, S., Heatubun, C. D., Lewis, C. E., Maturbongs, R. A., & Norup, M. V. (2006). *Dransfieldia* (Arecaceae) -A new palm genus from western New Guinea. *Systematic Botany*, 31, 61-69. <https://doi.org/10.1600/036364406775971705>

Barfod, A. S., & Heatubun, C. D. (2009). Two new species of *Licuala* Thunb. (Arecaceae: Coryphoideae) from North Moluccas and Western New Guinea. *Kew Bulletin*, 64, 553.  <https://doi.org/10.1007/s12225-009-9129-z>

Barrow, S. C. (1998). A monograph of *Phoenix* L.(Palmae: Coryphoideae). *Kew Bulletin*, 53, 513–575.

Bernal, R., & Borchsenius, F. (2010). Taxonomic novelties in *Aiphanes* (Palmae) from Colombia and Venezuela. *Caldasia*, 32, 117-127.

Bernal-González, R., & Henderson, A. (1986). A new species of *Socratea* (Palmae) from Colombia with notes on the genus. *Brittonia*, 38, 55-59. <https://doi.org/10.2307/2807419>

Borchsenius, F., & Bernal, R. (1996). *Aiphanes* (Palmae). *Flora Neotropica*, 1-94. <https://www.jstor.org/stable/4393869>.

Cascante, A. (2000). Additions to the genus *Bactris* (Arecaceae) of Mesoamerica. *Palms*, 44, 146-150.

Caxambu, M. G., Geraldino, H. C. L., Dettke, G. A., da Silva, A. R., & dos Santos, E. N. (2015). Palmeiras (Arecaceae) nativas no município de Campo Mourão, Paraná, Brasil. *Rodriguésia*, 66, 259-270. <https://doi.org/10.1590/2175-7860201566116>

Craft, P (2018). The Copernicias of Cuba. Retrieved from <https://www.palmnutpages.com/articles>.

de Granville, J. J. (2007). A new species of *Bactris* (Palmae) from French Guiana. *Brittonia*, 59, 354-356. <https://doi.org/10.1007/BF03159552>

de Nevers, G., & Henderson, A. (1988). A New *Calyptrogyne* (Palmae: Geonomeae) from Panama. *Systematic Botany*, 13, 428-431. https://doi.org/10.2307/2419303

Deble, L. P., & Marchiori, J. N. C. (2006). *Butia lallemantii*, uma nova Arecaceae do Brasil. *Balduinia*, 9, 1-3. <https://dx.doi.org/10.5902/2358198014032>

Deble, L. P., Marchiori, J. N. C., da Silva Alves, F., & de Oliveira-Deble, A. S. (2012). *Butia quaraimana* (Arecaceae), uma nova espécie para o Rio Grande do Sul (Brasil). *Balduinia*, 33,09-20. <https://periodicos.ufsm.br/balduinia/article/viewFile/13899/8681>

Dominy, N. J., Svenning, J. C., & Li, W. H. (2003). Historical contingency in the evolution of primate color vision. *Journal of Human Evolution*, 44, 25-45. <https://doi.org/10.1016/S0047-2484(02)00167-7>

Dowe, J. L. (2009). A taxonomic account of *Livistona* R. Br. (Arecaceae). *Gardens Bulletin Singapore*, 60, 185–344.

Dowe, J. L. (2010). Australian palms: biogeography, ecology and systematics. CSIRO Publishing, Collingwood, Australia.

Dowe, J. L., & Cabalion, P. (1996). A taxonomic account of Arecaceae in Vanuatu, with descriptions of three new species. *Australian Systematic Botany*, 9, 1-60. <https://doi.org/10.1071/SB9960001>

Dowe, J. L., & Ferrero, M. D. (2001). Revision of *Calyptrocalyx* and the New Guinea species of *Linospadix* (Linospadicinae: Arecoideae: Arecaceae). *Blumea-Biodiversity, Evolution and Biogeography of Plants*, 46, 207-251. <https://www.repository.naturalis.nl/record/525860>

Dransfield, J. (1979). A monograph of *Ceratolobus* (Palmae). *Kew Bulletin*, 34, 1-33+ii. <https://doi.org/10.2307/4117966>.

Dransfield, J. (1984). The genus *Areca* (Palmae: Arecoideae) in Borneo. *Kew Bulletin*, 1-22. <https://doi.org/10.2307/4107852>.

Dransfield, J., & Baker, W. J. (2003). An account of the Papuasian species of *Calamus* (Arecaceae) with paired fruit. *Kew Bulletin*, 371-387. https://doi.org/10.2307/4120621

Dransfield, J., Rakotoarinivo, M., Baker, W. J., Bayton, R. P., Fisher, J. B., Horn, J. W., ... & Metz, X. (2008). A new Coryphoid palm genus from Madagascar. *Botanical Journal of the Linnean Society*, 156, 79-91.  <https://doi.org/10.1111/j.1095-8339.2007.00742.x>

Dransfield, J., Uhl, N. W., Asmussen, C. B., Baker, W. J., Harley, M. M., & Lewis, C. E. (2008). Genera Palmarum - the evolution and classification of palms. Royal Botanic Gardens, Kew, London.

Ehara, H. (2018). Genetic variation and agronomic features of *Metroxylon* palms in Asia and Pacific. In *Sago Palm* (pp. 45-59). Springer, Singapore.

Essig, F. B. (1977). A systematic histological study of palm fruits. I. The *Ptychosperma* alliance. *Systematic Botany*, 2, 151-168. <https://doi.org/10.2307/2418257>

Essig, F. B. (1978). A revision of the genus *Ptychosperma* Labill.(Arecaceae). *Allertonia*, 1, 415-478. <https://www.jstor.org/stable/23186209>

Essig, F. B. (1980). The genus *Orania* Zipp (Arecaceae) in New Guinea. Lyonia, 1, 211-233.<https://hdl.handle.net/10125/10730>

Essig, F. B., & Hernandez, N. (2002). A systematic histological study of palm fruits. V. Subtribe Archontophoeniciae (Arecaceae). *Brittonia*, 54, 65-7. https://doi.org/10.1663/0007

Essig, F. B., Bussard, L., & Hernandez, N. (2001). A systematic histological study of palm fruits. IV. Subtribe Oncospermatinae (Arecaceae). *Brittonia*, 53, 466-471. <https://doi.org/10.1007/BF02809802>

Essig, F. B., Manka, T. J., & Bussard, L. (1999). A systematic histological study of palm fruits. III. Subtribe Iguanurinae (Arecaceae). *Brittonia*, 51, 307-325. <https://doi.org/10.2307/2666611>

Evans, R. J. (1995). Systematics of *Cryosophila* (Palmae). *Systematic Botany Monographs*, 46, 1- [https://doi.org/70. 10.2307/25027854](https://doi.org/70.%2010.2307/25027854)

Fernando, E. S. (1990). The genus *Heterospathe* (Palmae: Arecoideae) in the Philippines. *Kew Bulletin*, 45, 219-234. <https://doi.org/10.2307/4115681>

Galeano, G., & Bernal, R. (2010). Palmas de Colombia: guía de campo. CO-BAC, Bogotá.

Gari, N. M. (2005). Studies on Bali salak cultivars (*Salacca zalacca var. amboinensis*) (Arecaceae) (Doctoral dissertation, James Cook University). Retrieved from <http://researchonline.jcu.edu.au/1329/>.

Glassman, S. F. (1999). A taxonomic treatment of the palm subtribe Attaleinae (tribe Cocoeae). Urbana: University of Illinois Press, Illinois.

Heatubun, C. D. (2002). A monograph of *Sommieria* (Arecaceae). *Kew Bulletin*, 57, 599-611. https://doi.org/10.2307/4110988

Heatubun, C. D. (2011). Seven new species of *Areca* (Arecaceae). *Phytotaxa*, 28, 6-26.

Heatubun, C. D., Baker, W. J., Mogea, J. P., Harley, M. M., Tjitrosoedirdjo, S. S., & Dransfield, J. (2009). A monograph of *Cyrtostachys* (Arecaceae). *Kew Bulletin*, 64, 67-94. <https://doi.org/10.1007/s12225-009-9096-4>

Heatubun, C. D., Zona, S., & Baker, W. J. (2014). Three new genera of Arecoid palm (Arecaceae) from eastern Malesia. *Kew Bulletin*, 69, 1–18. <https://doi.org/10.1007/s12225-014-9525-x>

Henderson , A. (2011). A revision of *Geonoma* (Arecaceae). *Phytotaxa* ,17, 1-271.

Henderson, A. (2007). A revision of *Wallichia* (Palmae). *Taiwania*, 52, 1-11.

Henderson, A. (1990). Arecaceae part I. Introduction and the Iriarteinae. *Flora Neotropica*, 53, 1-101.

Henderson, A. (1995). The Palms of the Amazon. Oxford University Press, New York.

Henderson, A. (2000). *Bactris* (Palmae)*. Flora Neotropica Monograph*, 79, 1-181.

Henderson, A. (2002). Evolution and Ecology of Palms. The New York Botanical Garden Press, Bronx.

Henderson, A. (2005). A multivariate study of *Calyptrogyne* (Palmae). *Systematic Botany*, 30, 60-83.

Henderson, A. (2006). A New Species of *Arenga* (Palmae). *Taiwania,* 51*,* 298-301.

Henderson, A. (2012). A revision of *Pholidostachys* (Arecaceae). *Phytotaxa*, *43*, 1-48. <http://www.mapress.com/phytotaxa/content/2012/f/pt00043p048.pdf>

Henderson, A. J. (2004). A multivariate analysis of *Hyospathe* (Palmae). *American Journal of Botany*, 91, 953-965. <http://bsapubs.onlinelibrary.wiley.com/doi/pdf/10.3732/ajb.91.6.953>

Henderson, A. J. (2009). Palms of Southern Asia. Princeton Univ. Press, Princeton, NJ.

Henderson, A. J., & Bacon, C. D. (2011). *Lanonia* (Arecaceae: Palmae), a new genus from Asia, with a revision of the species. *Systematic Botany*, 36, 883-895.  <https://doi.org/10.1600/036364411X604903>

Henderson, A. J., & Martins, R. (2002). Classification of specimens in the *Geonoma stricta* (Palmae) complex: the problem of leaf size and shape. *Brittonia*, 54, 202–212.

Henderson, A., & Dung, N.Q. (2010). Notes on rattans (Arecaceae) from Vietnam. *Phytotaxa*, 8, 25–33.

Henderson, A., Ban, N. K., & Thanh, B. V. (2010). New species of *Areca*, *Pinanga*, and *Licuala* (Arecaceae) from Vietnam. *Phytotaxa*, 8, 34-40.

Henderson, A., Galeano, G., & Bernal, R. (1995). Field Guide to the Palms of the Americas. Princeton University Press, Princeton, New Jersey.

Hendra, M. (2002). Notes on a new record and a new species of *Pinanga* (Arecaceae) from Sumatra (Indonesia). *Floribunda,* 2*,* 1-8.

Herrera, J. (1987). Flower and fruit biology in southern Spanish Mediterranean shrublands. *Annals of the Missouri Botanical Garden*, 74, 69-78. <https://doi.org/10.2307/2399263>

Herrera, J. (1989). On the reproductive biology of the dwarf palm, *Chamaerops humilis* in southern Spain. *Principes*, 33, 27-32.

Hodel, D. R. (1992). *Chamaedorea* palms: The species and their cultivation. International Palm Society.

Hodel, D. R. (2007). A review of the genus Pritchardia. Palms, 51, S1-S53.

Hodel, D. R., & Marcus, J. (2011). *Cyphosperma naboutinense*, a New Species from Fiji. *Palms*, 55, 176. <https://www.palms.org/palmsjournal/2011/vol55n4p176-182.pdf>

Hsu, B., Coupar, I. M., & Ng, K. (2006). Antioxidant activity of hot water extract from the fruit of the Doum palm, *Hyphaene thebaica*. *Food Chemistry*, 98, 317-328. <https://doi.org/10.1016//j.foodchem.2005.05.077>

Jeanson, M. L., & Guo, L. (2011). *Arenga longicarpa*, a poorly known species from South China. *Palms*, 55, 122. <http://www.palms.org/palmsjournal/2011/vol55n3p122-130.pdf>

Jermy, A. C. (1980). Notulae et Novitates Muluenses: No. 1. *Botanical Journal of the Linnean Society*, 81, 1-46.  <https://doi.org/10.1111/j.1095-8339.1980.tb00940.x>

Kahn, F. (2008). El género *Astrocaryum* (Arecaceae). *Revista Peruana de Biología*, 15, 31-48.

Kahn, F., & De Granville, J. J. (1998). *Astrocaryum minus*, rediscovered in French Guiana. *Principes*, 42, 171-178. <https://www.palms.org/principes/1998/vol42n3p171-178.pdf>

Kahn, F., & Ferreira, E. J. L. (1995). A new species of *Astrocaryum* (Palmae) from Acre, Brazil. *Candollea*, 50, 321-328.

Keim, A. P., & Dransfield, J. (2012). A monograph of the genus *Orania* (Arecaceae: Oranieae). *Kew Bulletin*, 67, 127-190.

Kueffer, C., Kronauer, L., & Edwards, P. J. (2009). Wider spectrum of fruit traits in invasive than native floras may increase the vulnerability of oceanic islands to plant invasions. *Oikos*, 118, 1327-1334. <https://doi.org/10.1111/j.1600-0706.2009.17185.x>

Lestari, R., Ebert, G., & Huyskens-Keil, S. (2011). Growth and physiological responses of *Salak* cultivars (*Salacca zalacca* (Gaertn.) Voss) to different growing media. *Journal of Agricultural Science*, 3, 261. <http://dx.doi.org/10.5539/jas.v3n4p261>

Lim T. K., 2016, Edible Medicinal and Non-Medicinal Plants: Volume 11 Modified Stems, Roots and Bulbs, Springer International Publishing AG Switzerland, pp. 3-28.

Loo, A. H. (2011). Rediscovery in Singapore of *Salacca affinis* Griff, Arecaceae. *Nature in Singapore*, 4, 123-126. <http://lkcnhm.nus.edu.sg/app/uploads/2017/06/2011nis123-126.pdf>

Lorenzi, H. (2010). Brazilian Flora Arecaceae (Palms). Nova Odessa, Instituto Plantarum.

Ludwig, N., Lavergne, C., & Sevathian, J. C. (2010). Notes on the conservation status of Mauritian palms. *Palms*, 54, 77-93.

Machahua, M., Kahn, F., & Millán, B. (2014). Variabilidad vegetativa intra e interespecífica de *Astrocaryum chonta* y *A. javarense* (Arecaceae) en Jenaro Herrera, Loreto, Perú. *Revista Peruana de Biología*, 21, 139-144. <https://doi.org/10.15381/rpb.v21i2.9816>

Martins, R. C. (2012). A família Arecaceae (Palmae) no estado de Goiás: Florística e Etnobotânica. http://repositório.unb.br/handle/10482/12165

McClatchey, W. (1998). A new species of *Metroxylon* (Arecaceae) from Western Samoa. *Novon*, 8, 252-258. <https://doi.org/10.2307/3392014>

Mogea, J. P. (1980). The flabellate-leaved species of *Salacca* (Palmae). *Reinwardtia*, 9, 461-479.

Mónica, M. R., Borchsenius, F., & Blicher-Mathiesen, U. (1996). Notes on the biology and uses of the motacú palm (*Attalea phalerata*, Arecaceae) from Bolivia. *Economic Botany*, 50, 423-428. <https://doi.org/10.1007/BF02866525>

Moraes, M. (1996). *Allagoptera* (Palmae). *Flora Neotropica*, 73, 1-34. https://www.jstor.org/stable/4393875

Morici, C., & Pérez, R. V. (2006). *Coccothrinax torrida* (Arecaceae), a new species from southeastern Cuba. *Brittonia*, 58, 189-193. <https://doi.org/10.1663/0007196X>

Noblick, L. R. (2017). A revision of the genus *Syagrus* (Arecaceae). *Phytotaxa*, 294, 1–262. <https://doi.org/10.11646/phytotaxa.294.1.1>

Noblick, L. R., & Lorenzi, H. (2010). New *Syagrus* species from Brazil. *Palms*, 54, 18.

Oren, R., Zimmermann, R., & Terbough, J. (1996). Transpiration in upper Amazonia floodplain and upland forests in response to drought‐breaking rains. *Ecology*, 77, 968-973. <https://doi.org/10.2307/2265517>

Petoe, P., Cámara-Leret, R., & Baker, W. J. (2018). A monograph of the *Hydriastele wendlandiana* group (Arecaceae: *Hydriastele*). *Kew Bulletin*, 73, 17. <https://doi.org/10.1007/s12225-018-9736-7>

Quero, H. J. (2000). *Brahea sarukhanii*, a new species of palm from Mexico. *Palms*, 44, 109-113.

Quigley, D. T. G., Gainey, P. A., Pyne, A., & Hill, R. (2017). Prickly palm *Acrocomia* spp. (Arecaceae) endocarps: first records from Irish waters and a review of NW European records. *New Journal of Botany*, 7, 51-56. https://doi.org/[10.1080/20423489.2017.1354470](https://doi.org/10.1080/20423489.2017.1354470)

Rakotoarinivo, M., & Dransfield, J. (2010). New species of *Dypsis* and *Ravenea* (Arecaceae) from Madagascar. *Kew Bulletin*, 65, 279-303. <https://doi.org/10.1007/s12225-010-9210-7>

Rustiami, H. (2009). Two new species of *Daemonorops* from Sulawesi. *Reinwardtia*, 13, 25-30. https://doi.org/[10.14203/reinwardtia.v13i1.432](http://dx.doi.org/10.14203/reinwardtia.v13i1.432)

Rustiami, H., Mogea, J. P., & Tjitrosoedirdjo, S. S. (2011). Revision of the rattan genus *Daemonorops* (Palmae: Calamoideae) in Sulawesi using a phenetic analysis approach. *Gardens’ Bulletin Singapore*, 63, 17–30. <https://www.nparks.gov.sg/sbg/research/publications/gardens-bulletin-singapore/-/media/sbg/gardens-bulletin/4-4-63-1-2-02-y2011-v63-p1-p2-gbs-pg-17.pdf>

Sanín, M. J., & Galeano, G. (2011). A revision of the Andean wax palms, *Ceroxylon* (Arecaceae). *Phytotaxa*, 34, 1-64. <http://dx.doi.org/10.11646/phytotaxa.34.1.1>

Salzman, V. T., & Judd, W. S. (1995). A revision of the Greater Antillean species of *Bactris* (Bactridinae: Arecaceae). *Brittonia*, 47, 345-371. <https://doi.org/10.2307/2807563>

Saw, L. G. (2012). A revision of *Licuala* (Arecaceae, Coryphoideae) in Borneo. *Kew Bulletin*, 67, 577-654. <https://doi.org/10.1007/s12225-012-9414-0>

Soares, K. P., Longhi, S. J., Witeck Neto, L., & Assis, L. C. D. (2014). Palms (Arecaceae) from Rio Grande do Sul, Brazil. *Rodriguésia*, 65, 113-139. <https://rodriguesia.jbrj.gov.br>.

Stauffer, F. W., Asmussen, C. B., Henderson, A., & Endress, P. K. (2003). A revision of *Asterogyne* (Arecaceae: Arecoideae: Geonomeae). *Brittonia*, 55, 326. https://doi.org/10.1663/0007-196X(2003)055[0326:AROAAA]2.0.CO2

Sunderland, T. C. (2012). A taxonomic revision of the rattans of Africa (Arecaceae: Calamoideae). *Phytotaxa*, 51, 1-76.

Trudgen, M. S., & Baker, W. J. (2008). A revision of the *Heterospathe elegans* (Arecaceae) complex in New Guinea. *Kew Bulletin*, 63, 639-647. <https://doi.org/10.1007/s12225-008-9063-5>

Van Valkenburg, J. L. C. H., & Sunderland, T. C. H. (2008). A revision of the genus *Podococcus* (Arecaceae). *Kew Bulletin*, 63, 251-260. <https://doi.org/10.1007/s12225-008-9037-7>

Van Valkenburg, J. L. C. H., Sunderland, T. C. H., & Couvreur, T. L. P. (2008). A revision of the genus *Sclerosperma* (Arecaceae). *Kew Bulletin*, 63, 75-86. <https://doi.org/10.1007/s12225-007-9002-x>

Vianna, S. A. (2017). A new species of *Acrocomia* (Arecaceae) from Central Brazil. *Phytotaxa*, *314*, 45-54. https://dx.doi.org/10.11646/phytotaxa.314.1.2

Zona, S. (1990). A monograph of *Sabal* (Arecaceae: Coryphoideae). *Aliso: A Journal of Systematic and Evolutionary Botany*, 12, 583-666. <https://doi.org/10.5642/aliso.19901204.02>

Zona, S. (2005). A revision of *Ptychococcus* (Arecaceae). *Systematic Botany*, 30, 520-529.  <https://doi.org/10.1600/0363644054782152>.

**Table S3.** Comparative fit of two alternative evolutionary models for covariation between palm fruit size and height (i.e. fruit diameter _log10_ ~ height _log10_) before correlation analysis. **BM**: Brownian-Motion model and **OU**: Ornstein-Uhlenbeck model. **AICc**: Akaike information criterion corrected for sample-size; **∆AIC**: difference in Akaike information criterion, and **AIC_w_**: weighted Akaike information criterion. Model fit was assessed using AICw. In bold, the best-fitting model supported by the data (AICw = 1).

| **Models** | **∆AIC** | **AIC_C_** | **AIC_w_** |
| --- | --- | --- | --- |
| OU | 0.000 | 771.566 | 1 |
| BM | 904.634 | 1676.200 | 0 |


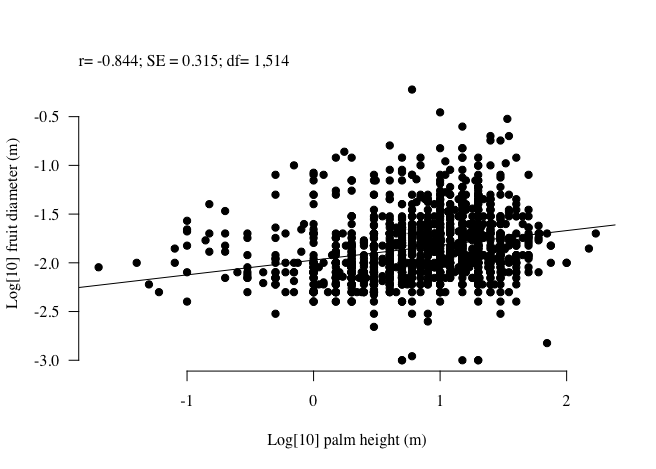
**Figure S5**. Phylogenetic generalized least squares (pGLS) correlation between palm fruit diameter (log 10) and height (log 10) for 1,516 species (which information on both traits was available) with its respective value and standard error (**SE**). To correlation test, we used the OU covariate matrix, which was the best-fitted covariance matrix for data structure (see Table S1 above).
